# Supplementary material for: Striatum-projecting prefrontal cortex neurons support working memory maintenance
Source: Nat Commun. 2023 Nov 2;14:7016. doi: 10.1038/s41467-023-42777-3 (PMC10622437; doi:10.1038/s41467-023-42777-3)
Supplement: Supplementary file 1 — Supplementary Information [file 41467_2023_42777_MOESM1_ESM.pdf]

Striatum-projecting prefrontal cortex neurons support working memory maintenance

Maria Wilhelm[1,2,6], Yaroslav Sych[1,7], Aleksejs Fomins[1,2], José Luis Alatorre Warren[1,8], Christopher Lewis[1], Laia Serratosa Capdevila[1], Roman Boehringer[3], Elizabeth A. Amadei[3], Benjamin Grewe[2,3,4], Eoin C. O'Connor[5], Benjamin J. Hall[5,9], Fritjof Helmchen[1,2,4]*

[1]Brain Research Institute, University of Zurich, 8057 Zurich, Switzerland.

[2]Neuroscience Center Zurich, University of Zurich and ETH Zurich, 8057 Zurich, Switzerland.

[3]Institute of Neuroinformatics, University of Zurich and ETH Zurich, 8057 Zurich, Switzerland.

[4]University Research Priority Program (URPP) Adaptive Brain Circuits in Development and Learning (AdaBD), University of Zurich, Zurich, Switzerland

[5]Neuroscience & Rare Diseases, Roche Pharma Research and Early Development, Roche Innovation Center Basel, F. Hoffmann-La Roche Ltd, Basel, Switzerland.

[6]Present address: Institute for Neuroscience, ETH Zurich, 8057 Zurich, Switzerland.

[7]Present address: Institute of Cellular and Integrative Neuroscience, CNRS, University of Strasbourg, Strasbourg, France.

[8]Present address: Center for Lifespan Changes in Brain and Cognition, University of Oslo, Oslo 0317, Norway.

[9]Present address: Circuit Biology Department, H. Lundbeck A/S, Valby, Denmark.

These authors contributed equally: Maria Wilhelm, Yaroslav Sych

*email: helmchen@hifo.uzh.ch

Supplementary information

This PDF file contains Supplementary Figures 1-15 with legends.

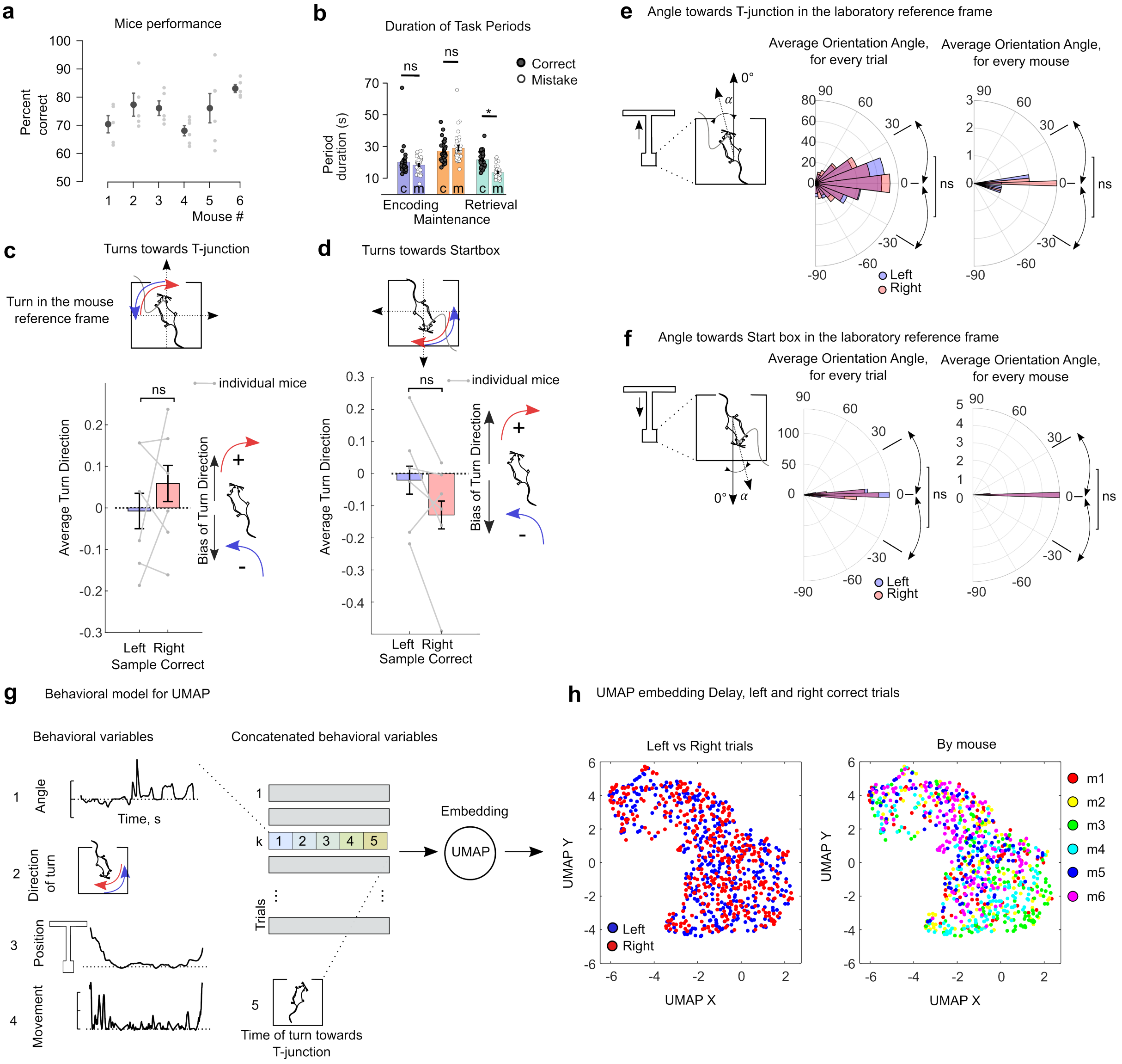

Supplementary Figure 1. Task performance and detailed analysis of behavioral variables during the maintenance period of the DNMTP T-maze task. (**a**) Average task performance across all expert mice used in fiber photometry experiments. n = 5-6 sessions for each of the 6 mice. (**b**) Duration of three task periods (Encoding, Maintenance, and Retrieval marked by purple, orange, and cyan respectively) for correct trials (black dots) and mistake trials (empty dots). Mean durations were not significantly different for encoding and maintenance periods (p(Ecorr vs Emist) = 1.0, p(Mcorr vs Mmist) = 1.0); the shorter retrieval period in the mistake trials (*p(Rcorr vs Rmist) = 0.03) reflects the absence of the reward collection phase (mistake trials were not rewarded); two-sided Wilcoxon rank sum test with Bonferroni correction, n = 32 sessions pooled across mice. (**c, d**) Trial-average number of turns during the maintenance period (-1 for every counterclockwise and +1 for every clockwise turn, overall reflecting the direction of the turn). Trial-average direction of turn was compared across all mice (n = 6) for 'Left' (all Left Sample Runs,' 405 trials) and 'Right' (all Right Sample Runs, 407 trials) correct trials. Turns were prevalent towards the T-junction and towards the opposite direction (start box). Therefore, we separately compared the trial-average number of turns towards these two directions. Neither clockwise turns nor counterclockwise turns during the maintenance period were predictive of future left vs. right turns at the T-junction (Wilcoxon rank sum test, c: p = 0.48 d: p = 0.48). (**e, f**) Mouse orientation angle (nose-to-tail base) relative to the axis of the main corridor (from start-box to T-junction) during the maintenance period is shown on the binned polar plot (left panel shows trial-average angle, all trials; right panel shows mouse-average orientation angle, n = 6 mice). We separated the orientation angle

into epochs when mice were facing towards the T-junction (e) or the opposite direction (f, start box). The orientation angle in the start box was not predictive of the direction of future left vs. right turn at the T-junction (Wilcoxon rank sum test, left plot for comparison by trials e: $p = 0.36$ f: $p = 0.58$; right plot for comparison by mouse e: $p = 0.7$ f: $p = 0.94$). (**g**) To account for more complex behavioral stereotypes or strategies, we calculated geometrical and behavioral variables potentially relevant for the DNMTP T-maze task (Methods): 1) the angle of mouse orientation (nose-to-tail base) relative to the axis of the T-maze main corridor (from start box to T-junction); 2) the number of turns during the maintenance period, with a turn defined as changing head direction from facing the T-junction to facing opposite or vice versa (separately counting clockwise and counter-clockwise turns). In addition, we quantified 3) the momentary position in the maze, 4) movement defined as positional changes of the nose (frame-to-frame displacement divided by frame interval, thus comprising both running speed and speed of head movements); and 5) the time of the first re-orienting turn towards the door after arrival in the start-box. All these behavioral variables could potentially explain either the future behavioral choice or the measured calcium signals. We reduced the dimensionality of this behavioral vector with UMAP embedding. (**h**) Left: UMAP embedding plane of behavioral variables. There were no differences for Left and Right trials (blue for Left Sample Run trials; red for Right Sample Run trials). Right: UMAP embedding plane trials labelled by mouse. Behavior gradually varied across mice, e.g., m3 and m4 had more similar behavior as compared to m5 and m6. When we inspected the UMAP embedding for every mouse separately we still could not find any separation of trials into left vs. right (data not shown). Data on all bar plots are shown as mean ± s.e.m.; two-sided Wilcoxon rank sum test with Bonferroni correction. ns, non-significant. Source data are provided as a Source Data file.

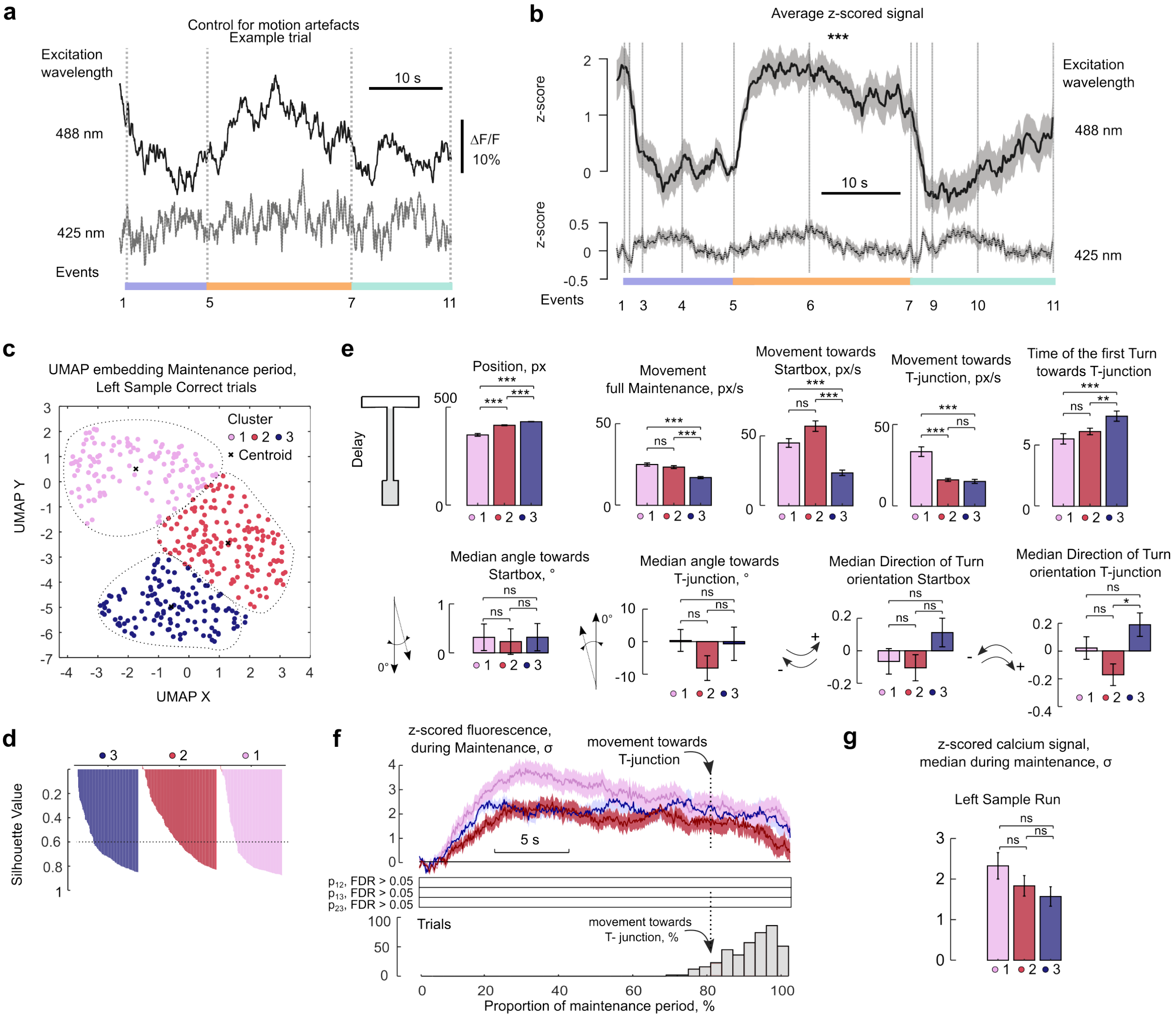

Supplementary Figure 2. Control for motion artefacts and contribution of motor-related behavioral variables to the photometry signal. (**a**) Single-trial example ΔF/F trace with 488-nm excitation (upper trace) and 425-nm excitation (lower trace; near-isosbestic for GCaMP6f). Traces were recorded simultaneously by delivering light through the same optical fiber and modulating each excitation wavelength at a different carrier frequency ("Methods"). (**b**) Average z-scored photometry signal recorded in 4 mice with dual-wavelength excitation. Control signals recorded with the 425-nm excitation wavelength had a significantly lower variance compared to the calcium-dependent signal recorded with the 488-nm excitation wavelength, confirming that the fluorescence was mostly emitted by the GCaMP6m sensor and excluding hemodynamic or motion-related artefacts as major sources of the maintenance signal. The mean z-score over the maintenance period was significantly higher for 488-nm compared to 425-nm excitation (p = 0.00019, paired Wilcoxon signed-rank test). (**c**) Clustering of behavioral data after dimensionality reduction (UMAP-embedding) with the *k-means* algorithm (MATLAB R2021). Out of 405 Left Sample Run trials: n(cluster 1) =127, n(cluster 2) =153 and n(cluster 3) = 125. (**d**) Silhouette values for data points within each cluster were not consistently high, suggesting that these were not distinct clusters but rather a continuum or multiple overlapping distributions. (**e**) Behavioral variables were different within each cluster (position: $p_{12} = 2\times10^{-13}$, $p_{13} = 6\times10^{-27}$, $p_{23} = 8\times10^{-9}$; movement full maintenance: p_{12} = 0.2, $p_{13} = 5\times10^{-11}$, $p_{23} = 3\times10^{-7}$; movement towards start box: p_{12} = 0.04, $p_{13} = 6\times10^{-11}$, $p_{23} = 9\times10^{-18}$; movement towards T-junction: $p_{12} = 2\times10^{-10}$, $p_{13} = 6\times10^{-13}$, p_{23} = 0.4; time of the first turn towards T-junction: p_{12} = 0.01, $p_{13} = 9\times10^{-5}$, p_{23} = 0.09; median angle towards start box: p_{12} = 0.08, p_{13} = 1.0, p_{23} = 0.6; median angle towards T-junction: p_{12} = 0.1, p_{13} = 2.1, p_{23} = 0.8; median direction of turn orientation start box: p_{12} = 0.2, p_{13} = 0.9, p_{23} = 0.6; median direction of turn orientation T-junction: p_{12} = 0.07, p_{13} = 1, p_{23} = 0.01; Wilcoxon rank sum test, Bonferroni corrected). (**f**)

Top: z-scored fluorescence signal during the maintenance period (data pooled across all 405 Left Sample Run correct trials from 6 mice). Signals were partitioned into three behaviourally identified clusters (mean and shaded area s.e.m. are shown for the photometry signal profile within each assigned cluster). Cluster 1 (pink) had transiently higher z-scored fluorescence (the bar below *$p<0.05$. Individual p-values corresponding to every time point (black line) are indicated in the Source Data file and were calculated by applying a two-sided Wilcoxon rank sum test to each time-bin comparison followed by false discovery rate correction (FDR, Benjamini-Yekutieli). Bottom: histogram of trials reflects the percent of time during the maintenance period when mice initiated movement towards the T-junction. Higher z-scored signals in cluster 1 did not temporally overlap with the high-amplitude movement during the delay period. However, signals correlated with movement towards T-junction ('movement' in **e**). (**g**) Median z-scored signals for the maintenance period did not show any dependence on the identity of behavioral clusters. Left Sample Run: $p_{12} = 0.6$, $p_{13} = 0.2$, $p_{23} = 1.0$; Wilcoxon rank sum test, Bonferroni corrected Solid lines represent mean, shaded area ± s.e.m. Data on all bar plots are shown as mean ± s.e.m. two-sided Wilcoxon rank sum test with Bonferroni correction. *$p<0.05$; **$p<0.01$; ***$p< 0.001$; ns, non-significant. Source data are provided as a Source Data file.

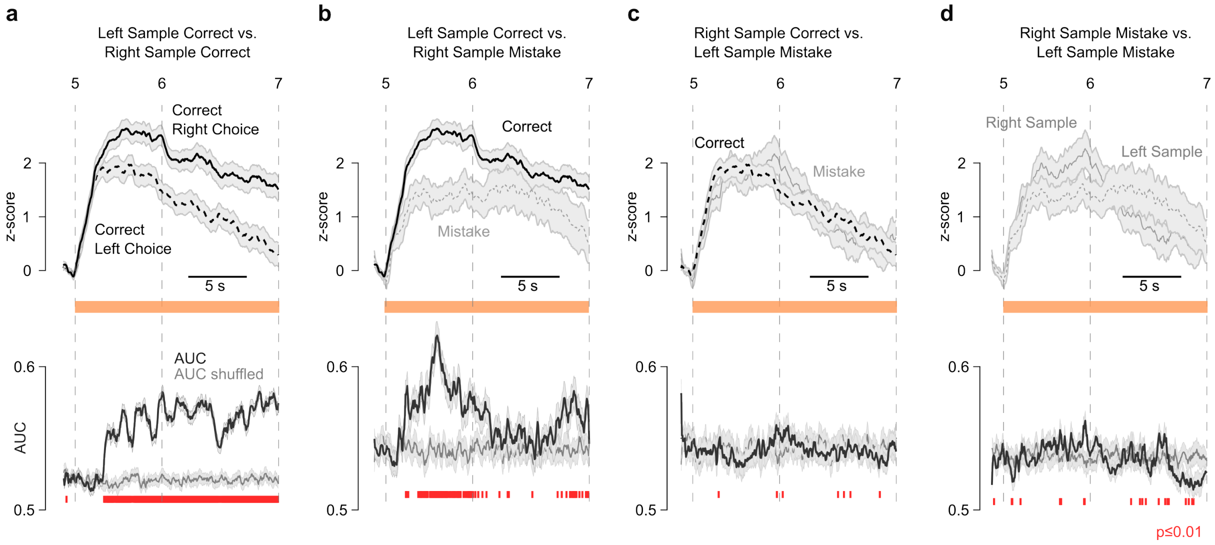

Supplementary Figure 3. mPFC→dmStr pathway activity during WM maintenance preceding different behavioural outputs. Top row: Average z-scored ΔF/F traces of mPFC→dmStr activity during the WM maintenance period, with each plot comparing two conditions (*n* = 6 mice). Dashed vertical lines indicate trial events defining the maintenance period. Bottom row: Area-under-the-curve (AUC) from the ROC analysis comparing the two conditions. (**a**) Comparison of correct choices (right or left) following left and right sample runs, respectively. (**b**) Comparison of right turns following left sample runs (correct choices) and right sample runs (mistakes). (**c**) Comparison of left turns following right sample runs (correct choices) and left sample runs (mistakes). (**d**) Comparison of incorrect choices (mistakes; left or right) following left and right sample runs, respectively. Throughout the figure, red markers at the bottom indicate time bins with significant (adjusted p-value ≤ 0.01) classification accuracy compared to trial-shuffled data (grey trace). Individual p-values corresponding to each red line (each time bin) are indicated in the Source Data file and were calculated by applying a two-sided Wilcoxon rank sum test to each time-bin comparison followed by FDR correction (Benjamini-Yekutieli). Solid lines represent mean, shaded area ± s.e.m. Source data are provided as a Source Data file.

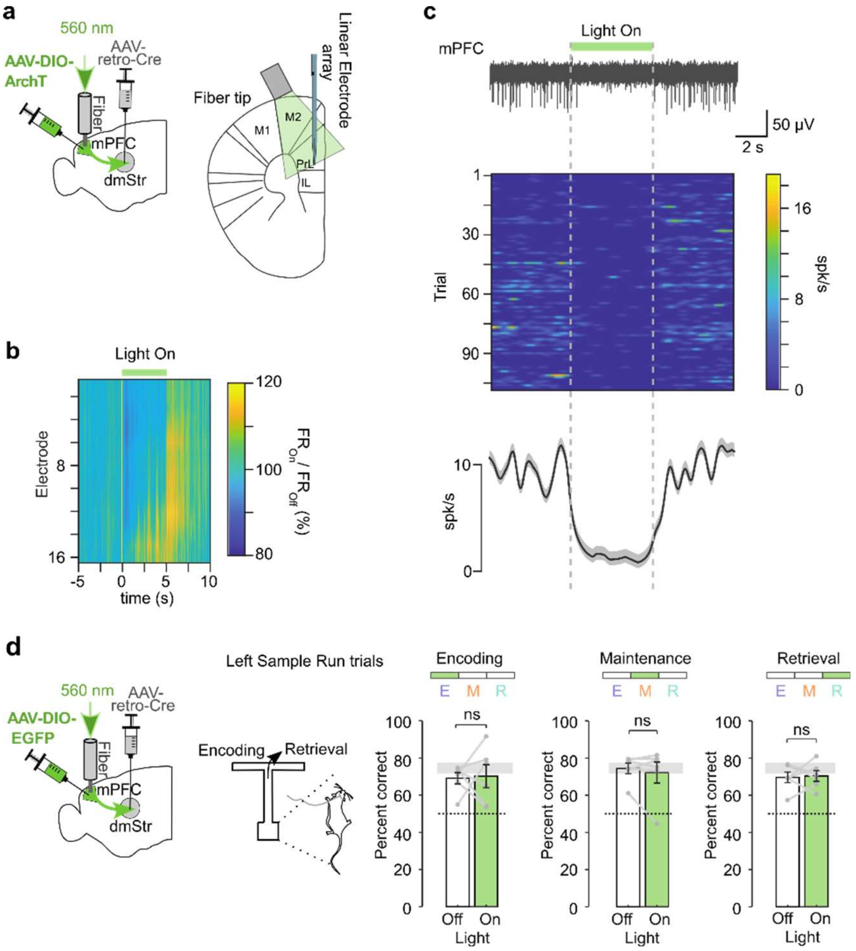

Supplementary Figure 4. Validation of pathway-specific optogenetic inhibition using ArchT. (**a**) Schematic of experimental design: mPFC→dmStr projection neurons were infected with a viral construct expressing Cre-dependent GFP-tagged ArchT. An optical fiber was placed on the brain surface for transient inhibition of neuronal activity with 561 nm light delivery. The linear probe was inserted vertically. (**b**) Light-evoked multi-unit activity along linear array in mPFC showing spatial distribution of suppression (16 sites linear array: top electrode – the most dorsal). **c**) Top: example cell in mPFC showing suppressed activity following light delivery to the surface of mPFC. Middle: average response, for *n* = 110 trials. Bottom: Spikes per second in a trial-wise light-modulation of the example cell. (**d**) To validate that the reduction of DNMTP task performance in ArchT experiments was due to the mPFC→dmStr silencing but not light illumination alone, we expressed EGFP fluorophore in the mPFC→dmStr pathway in the control cohort of mice (*n* = 6). No significant change in the task performance was observed during Light On condition as compared to Light Off during all periods of the task; *p*(E)= 1.0, *p*(M)= 1.0, *p*(R)= 1.0; two-sided Wilcoxon signed rank test with Bonferroni correction. Data on all bar plots are presented as mean ± s.e.m.. ns, non-significant. Source data are provided as a Source Data file.

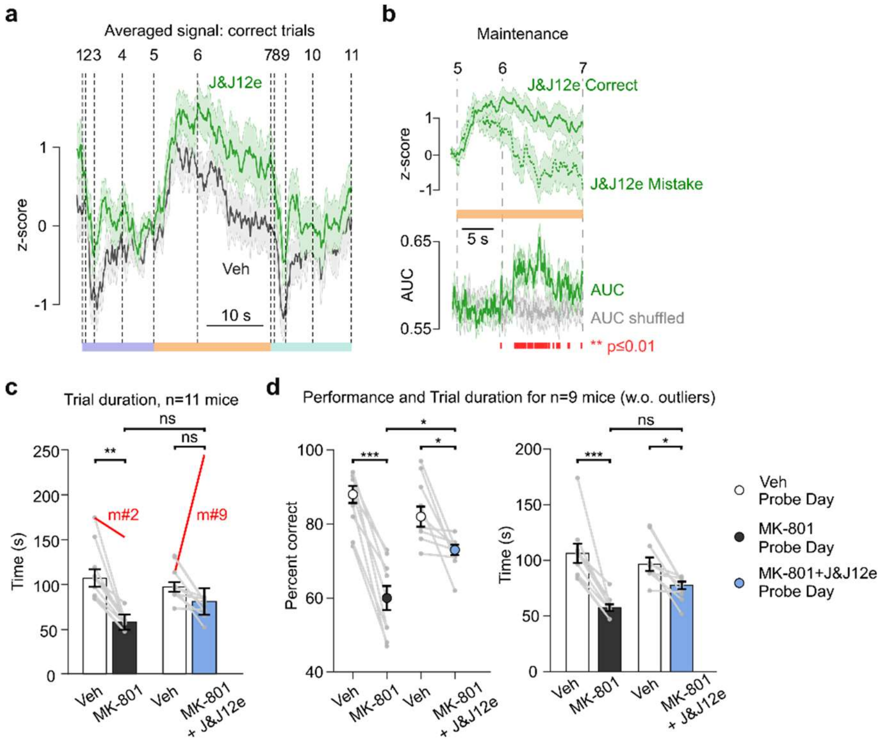

Supplementary Figure 5. Effect of HCN channels blockade on WM-related mPFC→dmStr activity and WM impairment induced by MK-801. (**a**) Resampled z-scored calcium traces for the J&J12e condition in green and vehicle (Veh) in black (*n* = 6 mice). Numbers correspond to the task events indicated in Fig 1. Solid lines represent average calcium transients, the shaded areas indicate s.e.m. (**b**) Top: With J&J12e application, mPFC→dmStr pathway calcium signals during the maintenance period are higher for correct trials (solid line) compared to mistake trials (dashed line). Bottom: Time course of correct vs. mistake classification based on AUC of an ROC analysis. Red markers at the bottom indicate time bins with significant (adjusted p-value ≤ 0.01) classification accuracy compared to trial-shuffled data (grey trace). Individual p-values corresponding to each red line (each time bin) are indicated in the Source Data file, and were calculated by applying a two-sided Wilcoxon rank sum test to each time-bin comparison followed by FDR correction (Benjamini-Yekutieli). Solid lines represent mean, shaded area ± s.e.m. (**c**) J&J12e attenuates MK-801-induced hyperlocomotion as indicated by trial durations (***p* = 0.0091 for the comparison between Veh and MK-801, all other exact p-values (ns p>0.05) are indicated in the Source data file. One-way ANOVA and Tukey post hoc test for multiple-group comparisons). Nearly all mice (9 out of 11) exhibited hyperlocomotion after receiving the MK-801. However, two mice (highlighted in red) displayed cataleptic-like behaviour (pausing in various places of the maze for several minutes), as has been reported previously (ref. 26). (**d**) Analysis of performance and trial duration for the various conditions restricted to the 9 mice that did not show cataleptic-like behaviour (performance: ****p* = $5.40\times10^{-7}$for Veh vs MK-801 comparison, **p* = 0.020 for Veh vs J&J12e, **p* = 0.013 for MK-801 vs J&J12e; trial duration: ****p* = 1.77×10^{-5} for Veh vs MK-801 comparison, **p* = 0.0168 for Veh vs J&J12e. All other exact p-values are indicated in the Source data file. One-way ANOVA and Tukey post hoc test for multiple-group comparisons). Data on all bar plots are shown as mean ± s.e.m. **p*<0.05; ***p*<0.01; ****p*< 0.001; ns, non-significant. Source data are provided as a Source Data file.

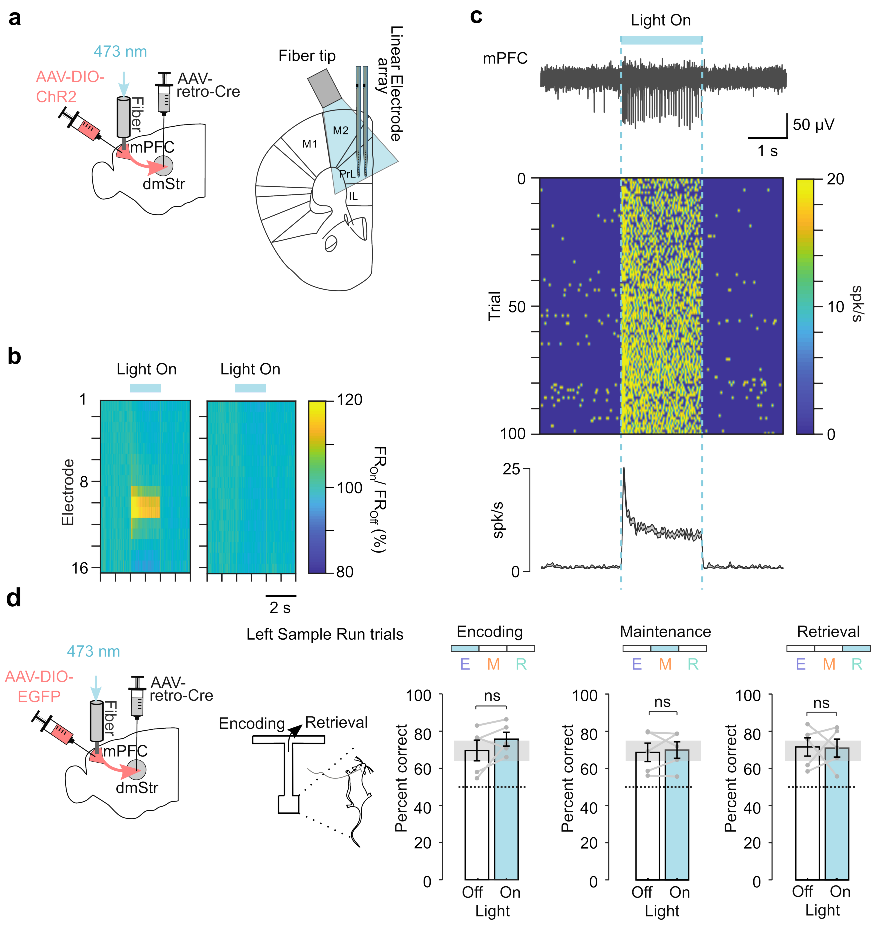

Supplementary Figure 6. Validation of pathway-specific optogenetic activation. (**a**) Scheme of ChR2 validation experiment: similar to Fig.5, mPFC→dmStr projecting neurons were infected by the Cre-dependent ChR2 virus injection, the optical fiber was placed on top of the brain surface for the transient activation of neuronal activity with 473-nm light delivery. (**b**) Light-evoked multi-unit activity along two linear arrays in mPFC showing spatial distribution of activation (2x16 site linear array: top electrodes are the most dorsal). (**c**) Top: Example cell in mPFC showing enhanced activity following light delivery to the surface of mPFC. Bottom: average response of the cell in (b, top) (*n* = 200 trials) c) Trial-wise light-modulation of the example cell (*n* = 200 trials). (**d**) To validate that the enhancement of DNMTP task performance in ChR2 activation of mPFC→dmStr + MK801 experiments was due to the activation but not light illumination alone, we expressed GFP fluorophore in the mPFC→dmStr pathway in the control cohort of mice (*n* = 5). No significant change in the task performance was observed during Light On condition as compared to Light Off during all periods of the task (p(E) = 0.56, p(M) = 1.0, p(R) = 1.0; two-sided Wilcoxon signed rank test with Bonferroni correction). Data on all bar plots are shown as mean ± s.e.m.; two-sided Wilcoxon rank sum test with Bonferroni correction. ns, non-significant. Source data are provided as a Source Data file.

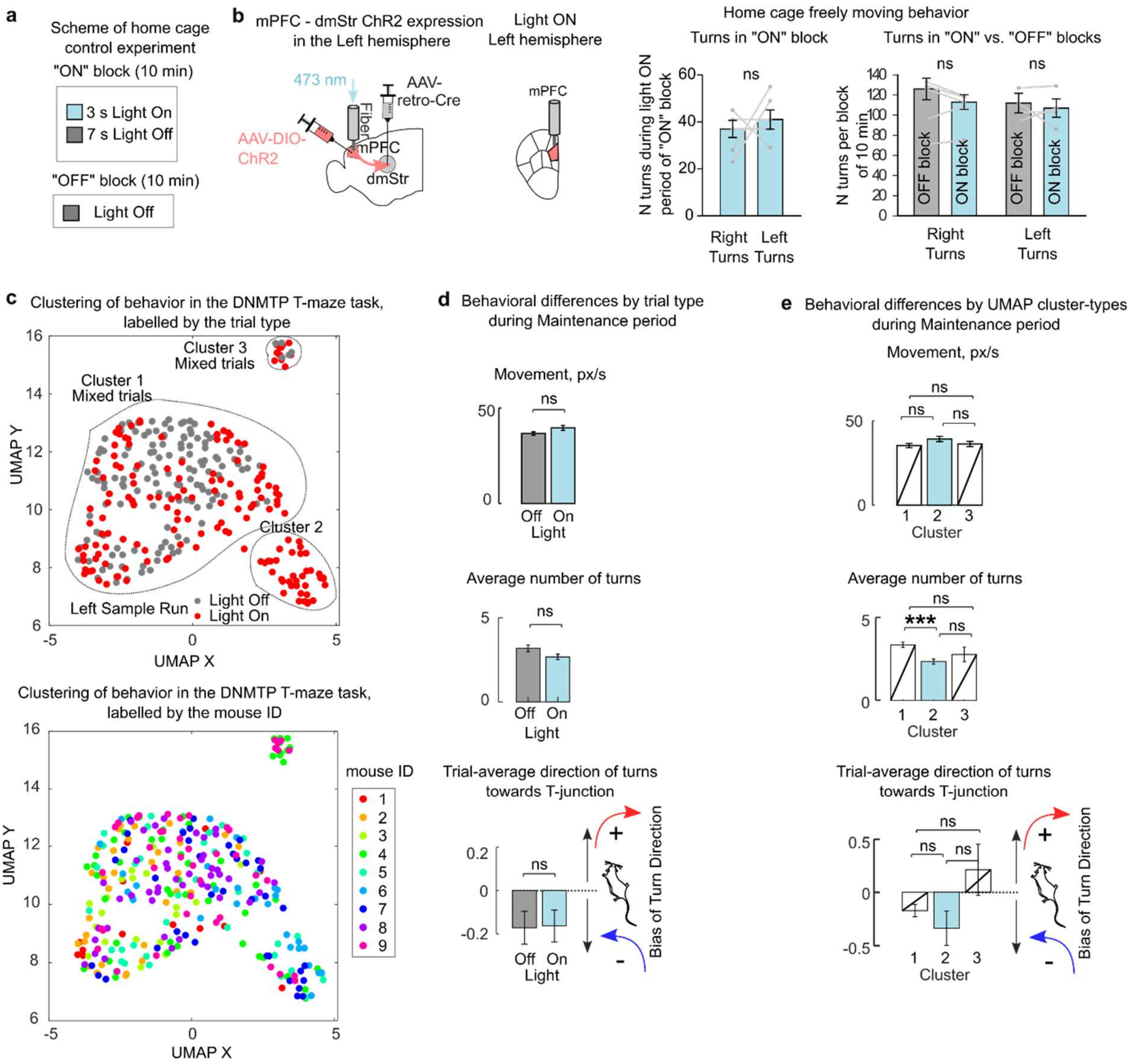

Supplementary Figure 7. Alternations of mouse behavior in the home cage and during the maintenance period of DNMTP T-maze task upon optogenetic activation of mPFC→dmStr pathway. (**a**) Design of home cage experiments to test for potential effects of ChR2 activation on motor behaviour (n = 5 mice with ChR2 expression). Mice were tested twice in their home cage: On the first day, explorative behaviour was monitored for 10 min with continuously alternating periods of 3-s laser light on and 7-s laser light off periods (ON-block), followed by a 10-min period without no laser illumination (OFF-block). On the second day, the order of the test blocks was reversed (first 10 min OFF-block, then 10 min ON-block). Data from both days was analyzed and pooled. (**b**) Left: We observed no significant difference in the number of right vs. left 90° turns inside the 3s activation period of the ON-block (p = 0.5, paired Wilcoxon signed-rank test). Right: The number of 90° turns within ON- vs. OFF-blocks did also not differ (p = 0.98 for the comparison between Right turns within OFF- and ON-blocks; p = 0.99 for the comparison between Left turns; all other exact p-values are indicated in the Source data file. One-way ANOVA and Tukey post hoc test for multiple-group comparisons; n = 5 mice). This analysis considered the entire 10-min duration per block and controls for potential long-lasting light-induced turning preferences that could have occurred during the 7-s light off period in the ON-block. (**c**) Dimensionality reduction of all behavioral variables (Supplementary Figure 1g, h) with UMAP. Top inset: UMAP-embedding labelled by the trial type. Cluster 1 and 3 contained trials from both Left

Sample correct Light On and Off trials (mixed within cluster trials). Cluster 2 contained only Light On trials (Laser On red, Laser Off grey). Bottom inset: UMAP-embedding labelled by the mouse id. Cluster 1 and 2 had contributions from multiple mice, while cluster 3 from 2 mice. Out of 307 trials: n(cluster 1) = 191, n(cluster 2) = 99 and n(cluster 3) = 17. (**d**) Movement ($p = 0.2$), average number of turns ($p = 1.0$), and direction of turns ($p = 1.0$) were averaged for every trial and compared in the Light On and Light Off group. (**e**) Same as d, but behavioral variables were compared for every cluster. In summary, overall motor movement ($p_{12} = 0.8$, $p_{13} = 1.5$, $p_{23} = 1.0$) and trial-average direction of turns towards T-junction ($p_{12} = 0.5$, $p_{13} = 0.9$, $p_{23} = 0.4$) did not change, however, we found subtle differences in the average number of turns (***$p_{12} = 2.5 \cdot 10^{-7}$, $p_{13} = 0.3$, $p_{23} = 0.1$) during Light On trials (two-sided Wilcoxon rank sum test, Bonferroni corrected). Data on all bar plots are shown as mean ± s.e.m.; ns, non-significant. Source data are provided as a Source Data file.

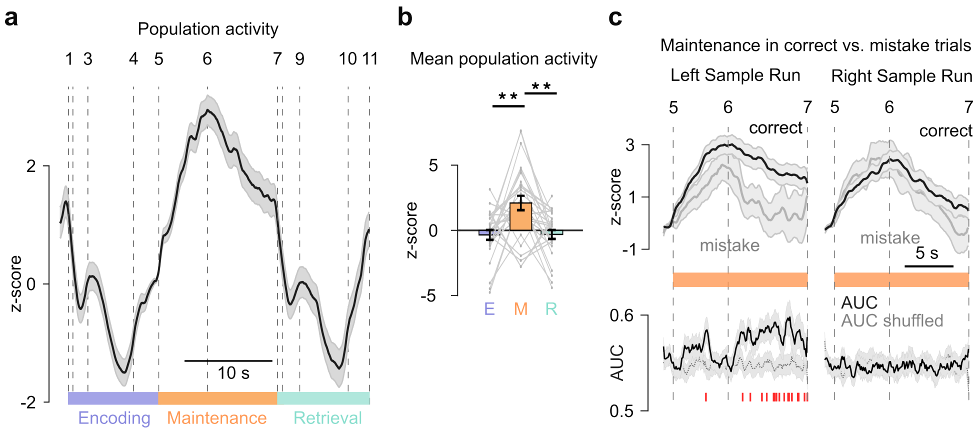

Supplementary Figure 8. Average neuronal population activity in the miniscope imaging data. (**a**) Mean task-related calcium signal across all identified neurons imaged with the miniscope (shaded area indicates s.e.m.). As for the fiber photometry data, neuronal ΔF/F traces were resampled and aligned to the task phases, z-scored, and finally averaged across sessions and mice (n = 6 mice; 3-5 sessions per mouse; 28-88 active neurons per mouse were longitudinally tracked across days). Dashed lines indicate task events for alignment as defined in Figure 1. (**b**) Average activity was higher during the maintenance period compared to encoding and retrieval periods (**p = 0.0010 for M vs E, **p = 0.0019 for M vs R, p = 0.97 for E vs R; n = 27 experimental sessions, one-way ANOVA and Tukey post hoc test for multiple-group comparisons). (**c**) Top: Mean z-scored calcium signals during the maintenance period for correct (black) and mistake (grey) trials, shown separately for periods following left sample runs (correct choice to the right, contra-lateral to the recording site) and right sample runs (correct choice to the ipsilateral side). Bottom: Time course of correct vs. mistake classification based on the area-under-the-curve (AUC) of an ROC analysis. Red markers at the bottom indicate time bins with significant (adjusted p-value ≤ 0.01) classification accuracy compared to trial-shuffled data (grey trace). Individual p-values corresponding to each red line (each time bin) are indicated in the Source Data file and were calculated by applying two-sided Wilcoxon rank sum test to each time-bin comparison followed by FDR correction (Benjamini-Yekutieli). Solid black lines represent mean, shaded area ± s.e.m. Data on the bar plot are shown as mean ± s.e.m. Source data are provided as a Source Data file.

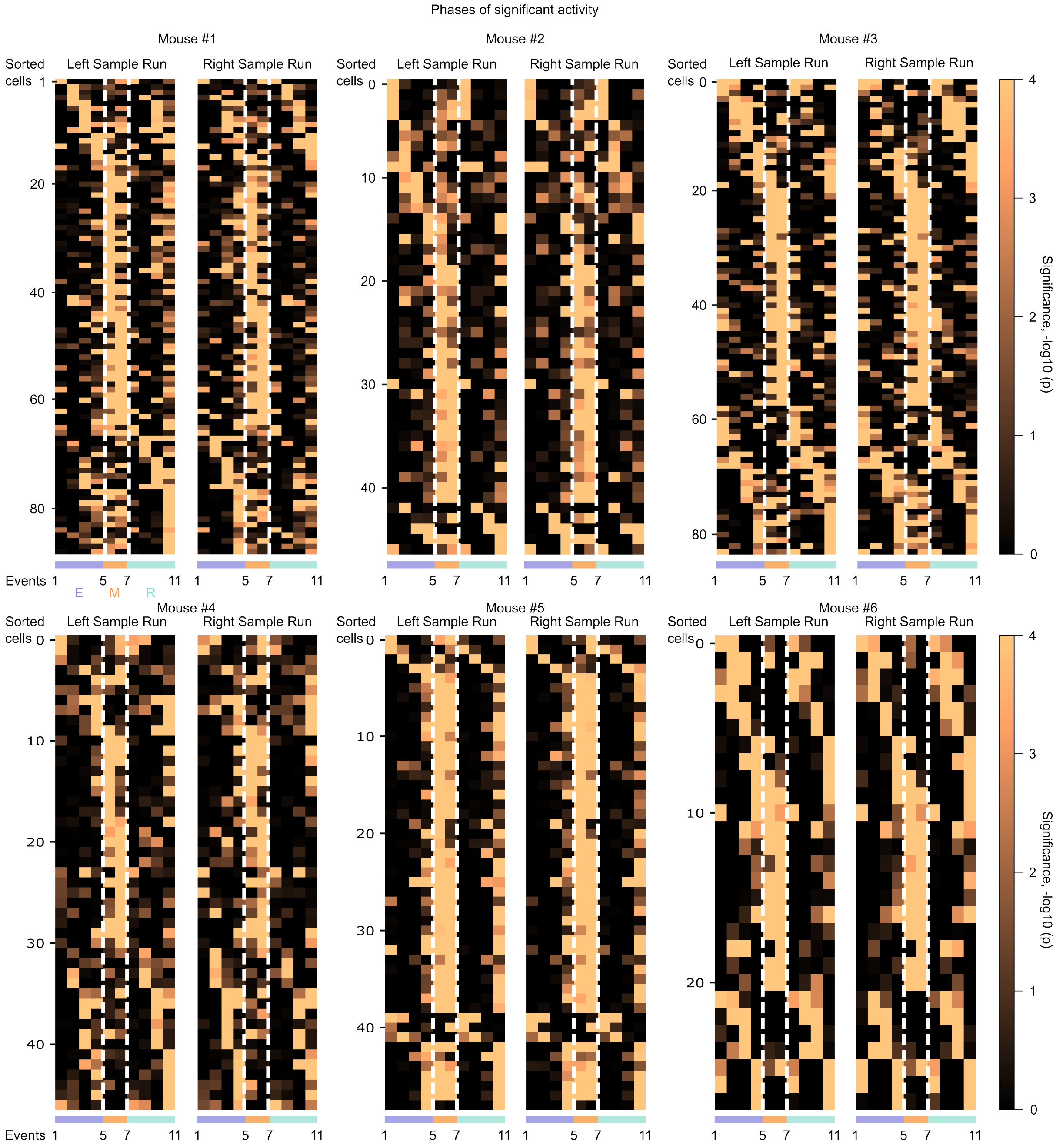

Supplementary Figure 9. Relative significance of single-cell activity during task phases for all individual mice. Significance level was calculated as $-\log 10(p)$, where p is the p-value of the Wilcoxon rank sum test, comparing the mean activity from the deconvolved ΔF/F traces for each neuron in a given phase to all other phases. Heatmaps show results separately for left and right sample runs. Single cells were sorted according to the phase with the highest significance level in the correct left sample trials. In all mice except for mouse #6, more cells were significantly more active in the maintenance period compared to encoding or retrieval periods (see quantification in Fig. 6f). Note that in some mice, e.g., m#1 and m#3, differences in the neuronal population activity during encoding and retrieval are apparent for left versus right turns. Number of longitudinally recorded neurons: 88 (m1), 46 (m2), 83 (m3), 47 (m4), 49 (m5), 28 (m6). Source data are provided as a Source Data file.

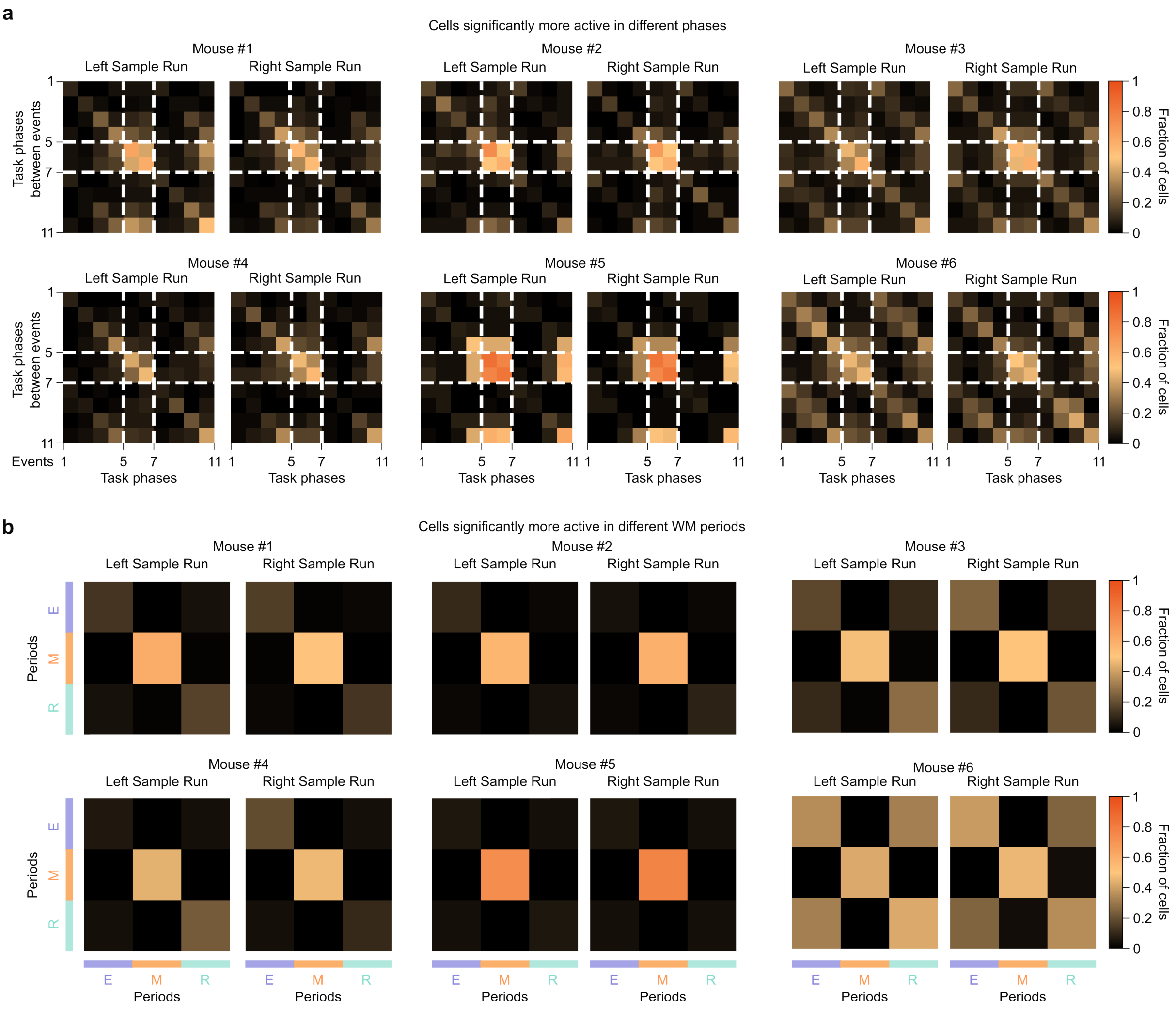

Supplementary Figure 10. Fraction of cells active in pairs of task phases and task periods for all individual mice. Matrix plots showing the fraction of neurons with significant activity in pairs of task phases (**a**) or task periods (**b**) for each individual mouse. Values on the diagonal represent the fraction of neurons significantly more active in the respective phase or period than in other phases/periods. Off-diagonal entries represent the fraction of neurons significantly more active in two different phases/periods simultaneously, compared to other phases/periods. Note the overlap of neuronal fractions that show significant activity in encoding and retrieval periods as well as in corresponding phases during these periods. Colored bars in (b) correspond to task periods (purple - encoding, orange - maintenance, cyan - retrieval). Except for mouse #6, the fraction of neurons active in the maintenance period is higher than in encoding or retrieval periods. Source data are provided as a Source Data file.

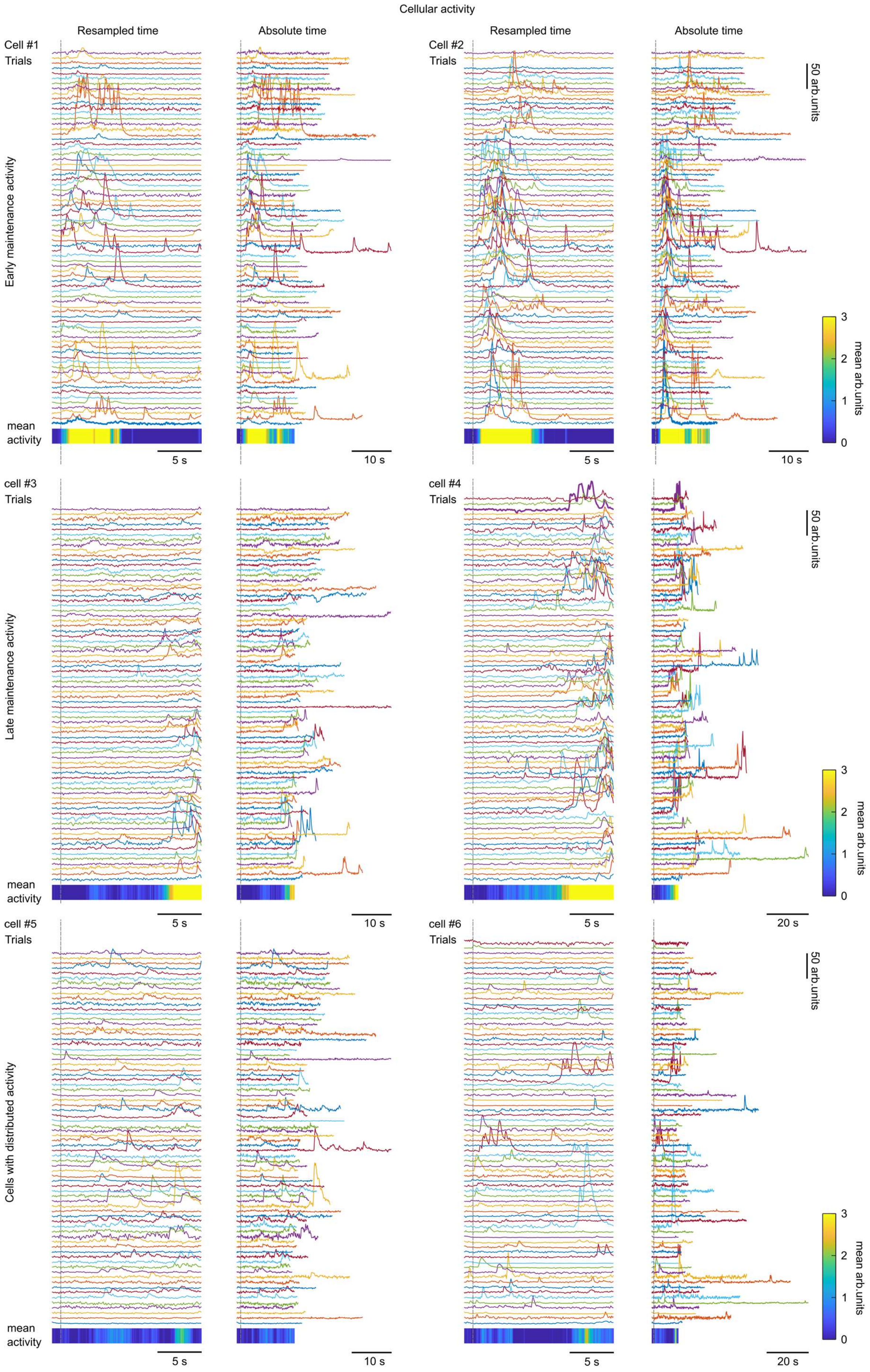

Cellular activity
Resampled time
Absolute time
Resampled time
Absolute time
Cell #1
Trials
Cell #2
Trials
50 arb.units
Early maintenance activity
mean activity
mean arb.units
3
2
1
0
5 s
10 s
5 s
10 s
cell #3
Trials
cell #4
Trials
Late maintenance activity
mean activity
5 s
10 s
5 s
20 s
cell #5
Trials
cell #6
Trials
Cells with distributed activity
mean activity
5 s
10 s
5 s
20 s

Supplementary Figure 11. Examples of cells displaying elevated activity during the maintenance period. For each cell, the raw output signal is plotted in arbitrary units (Similar to Fig. 6c left; Methods). The first column shows the cellular activity traces during the whole maintenance period (between events 5 and 7) resampled to the normalized time (median of the 5-7 duration over all trials of all sessions and mice). The heat map shows the mean activity. The second column shows the activity traces during maintenance in absolute time. Since the mice are freely moving, the time spent in the start box varies in some trials although all doors of the maze are open allowing mice to proceed to the choice run. Top row shows the example cells displaying activity in the early maintenance period, middle row shows cells active in the late maintenance period. The bottom row shows the cells, that were identified as delay cells, but the time point of their highest activity is not consistent from trial to trial.

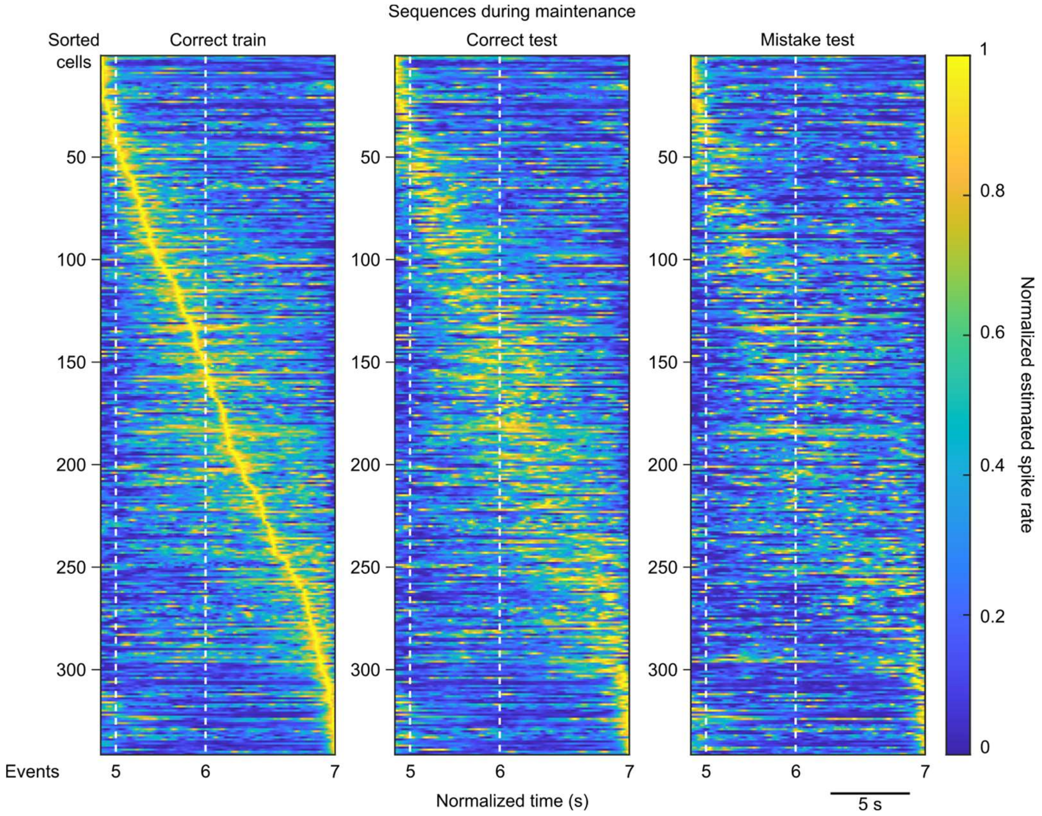

Supplementary Figure 12. Sequential activity of mPFC→dmStr projection neurons during the maintenance period. Normalized deconvolved neuronal signals during the two phases of the maintenance period were pooled for the miniscope data from all 6 mice (*n* = 341 neurons in total). Left: Mean signals for a randomly selected half of correct trials used as a training set. Middle: Mean signals for the remaining test set of correct trials. Right: Mean signals for mistake trials. In all plots, neurons are sorted according to the signal peak times in the training data. Source data are provided as a Source Data file.

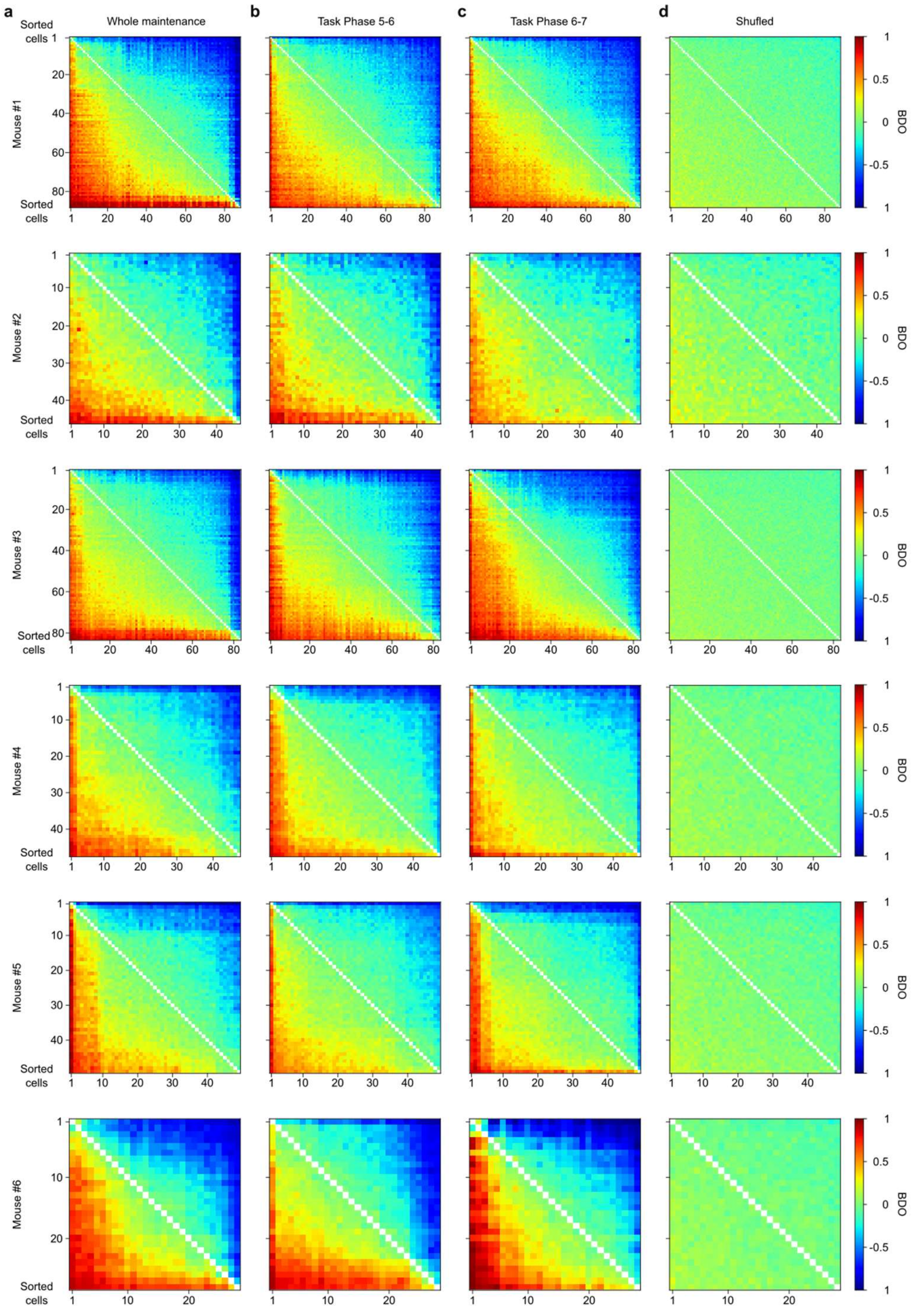

Supplementary Figure 13. Binary Directed Orderability matrixes for all individual mice. Each row shows the analysis for one mouse. (**a**) For the whole maintenance period. (**b**) For the first maintenance phase between events 5 and 6. (**c**) For the second maintenance phase between events 6 and 7. (**d**) For shuffled data as control. For each plot, the neurons (and thus rows and columns) are sorted according to the neuron's average *BDO* of the *BDO* corresponding to that plot, which, in general, results in a different order of neurons for different plots. Source data are provided as a Source Data file.

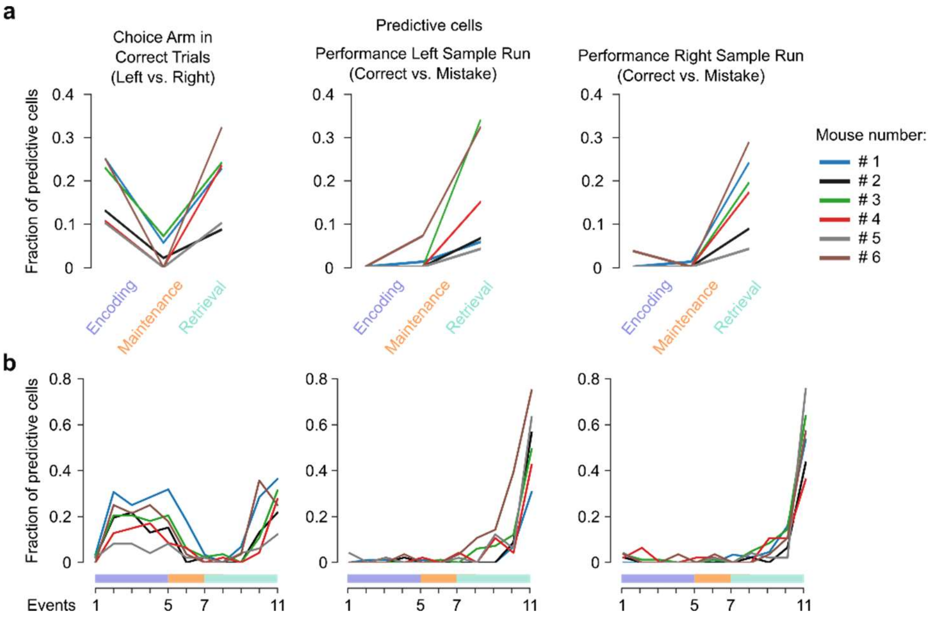

Supplementary Figure 14. Predictive power of mPFC→dmStr neuronal populations to classify left vs. right turning direction and correct vs. mistake choices across trial task time. (**a**) Fraction of cells predictive for choice direction (left vs. right turn and for performance (correct vs. mistake trials; separated for trials with left and right sample arm) in the different task periods. For a definition of predictive neurons see Methods. (**b**) Same as for (a) but for all 10 trial phases. Each colored line represents an individual mouse. For the turning direction, the fraction of cells predictive for encoding or retrieval periods is higher than for the maintenance period, which might correspond to the unique sub-population of cells active either in the right or left maze arm. Source data are provided as a Source Data file.

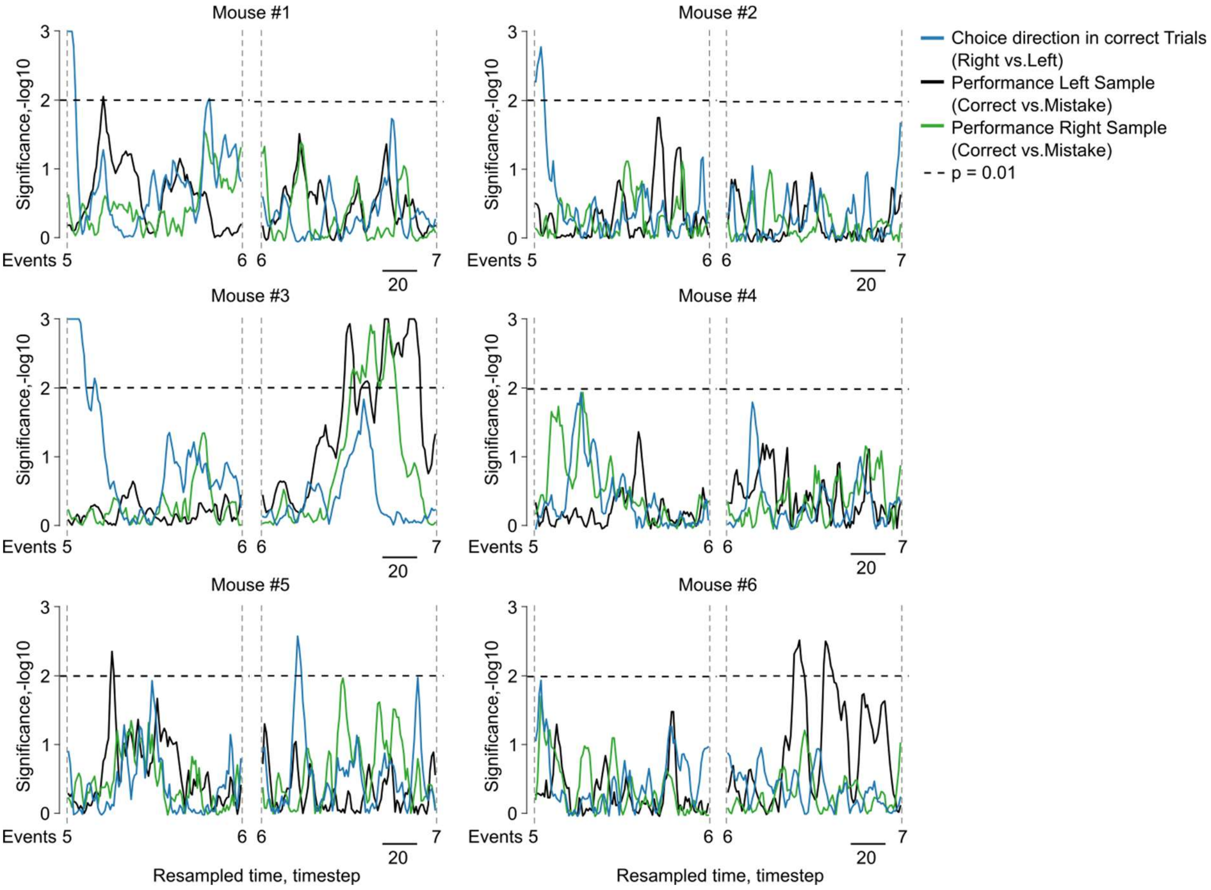

Supplementary Figure 15. Predictive power of mPFC→dmStr neuronal populations during the two phases of the maintenance period. We tested for population encoding of choice direction (left vs. right turn; blue traces) and of performance (correct vs. mistake trials; separated for trials with left sample arm [black traces] and right sample arm [green traces]). The analysis is based on the neuronal average distance between population vectors for the two considered conditions at each time bin of resampled trial time. We tested against data with shuffled condition labels (Methods). Dashed horizontal lines represent $-\log10(p)=2$, thus indicating chance level of p = 0.01. Each subpanel shows the analysis for an individual mouse. Note that mice #3 and #6 transiently reach significance during the second phase of the maintenance period for encoding performance when an upcoming contralateral (right) turn is correct. All traces during each maintenance phase have been resampled to 100 time bins for this analysis. Source data are provided as a Source Data file.
