# Supplementary material for: Striatum-projecting prefrontal cortex neurons support working memory maintenance
Source: Nat Commun. 2023 Nov 2;14:7016. doi: 10.1038/s41467-023-42777-3 (PMC10622437; doi:10.1038/s41467-023-42777-3)
Supplement: Supplementary file 3 — Reporting Summary [file 41467_2023_42777_MOESM3_ESM.pdf]

nature portfolio

Corresponding author(s): Fritjof Helmchen

Last updated by author(s): Oct 17, 2023

Reporting Summary

Nature Portfolio wishes to improve the reproducibility of the work that we publish. This form provides structure for consistency and transparency in reporting. For further information on Nature Portfolio policies, see our Editorial Policies and the Editorial Policy Checklist.

Statistics

For all statistical analyses, confirm that the following items are present in the figure legend, table legend, main text, or Methods section.

n/a	Confirmed	
☐	☒	The exact sample size (*n*) for each experimental group/condition, given as a discrete number and unit of measurement
☐	☒	A statement on whether measurements were taken from distinct samples or whether the same sample was measured repeatedly
☐	☒	The statistical test(s) used AND whether they are one- or two-sided<br>*Only common tests should be described solely by name; describe more complex techniques in the Methods section.*
☐	☒	A description of all covariates tested
☐	☒	A description of any assumptions or corrections, such as tests of normality and adjustment for multiple comparisons
☐	☒	A full description of the statistical parameters including central tendency (e.g. means) or other basic estimates (e.g. regression coefficient) AND variation (e.g. standard deviation) or associated estimates of uncertainty (e.g. confidence intervals)
☐	☒	For null hypothesis testing, the test statistic (e.g. *F*, *t*, *r*) with confidence intervals, effect sizes, degrees of freedom and *P* value noted<br>*Give P values as exact values whenever suitable.*
☒	☐	For Bayesian analysis, information on the choice of priors and Markov chain Monte Carlo settings
☐	☒	For hierarchical and complex designs, identification of the appropriate level for tests and full reporting of outcomes
☐	☒	Estimates of effect sizes (e.g. Cohen's *d*, Pearson's *r*), indicating how they were calculated

Our web collection on statistics for biologists contains articles on many of the points above.

Software and code

Policy information about availability of computer code

Data collection	Data was collected using custom-written code in Matlab and Inscopix software.
Data analysis	Data was analyzed custom-written scripts in Matlab (2016a,2019b, 2020b), DeepLabCut, and Python 3 and Inscopix Data Processing Software (version 1.2.1). Custom-written code is available on a GitHub repository (https://github.com/HelmchenLabSoftware/wilhelm-sych-tmaze-analysis). See our Code Availability statement.

For manuscripts utilizing custom algorithms or software that are central to the research but not yet described in published literature, software must be made available to editors and reviewers. We strongly encourage code deposition in a community repository (e.g. GitHub). See the Nature Portfolio guidelines for submitting code & software for further information.

Data

Policy information about availability of data

All manuscripts must include a data availability statement. This statement should provide the following information, where applicable:

- Accession codes, unique identifiers, or web links for publicly available datasets
- A description of any restrictions on data availability
- For clinical datasets or third party data, please ensure that the statement adheres to our policy

We now deposited our data on https://zenodo.org/records/8387632 and also provide SourceData files for all figures. See also our Code Availability statement.

Research involving human participants, their data, or biological material

Policy information about studies with human participants or human data. See also policy information about sex, gender (identity/presentation), and sexual orientation and race, ethnicity and racism.

Reporting on sex and gender	n/a
Reporting on race, ethnicity, or other socially relevant groupings	n/a
Population characteristics	n/a
Recruitment	n/a
Ethics oversight	n/a

Note that full information on the approval of the study protocol must also be provided in the manuscript.

Field-specific reporting

Please select the one below that is the best fit for your research. If you are not sure, read the appropriate sections before making your selection.

☒ Life sciences ☐ Behavioural & social sciences ☐ Ecological, evolutionary & environmental sciences

For a reference copy of the document with all sections, see nature.com/documents/nr-reporting-summary-flat.pdf

Life sciences study design

All studies must disclose on these points even when the disclosure is negative.

Sample size	For analysis of behavioural variables, photometric data, and the effect of opogenetic manipulations, the sample size (number of independent mice) was 6 to 11 mice. Because estimated effect sizes are between 1.0-1.5, this sample sizes matches the reuqired sample size for testing differences of the means. This sample size is also is in accordance with the number of animals used in previous similar neuroscientific studies. For analysis of neuronal population data, neurons were treated as independent variables (sample size, n = 341 neurons).
Data exclusions	No data was excluded from this study
Replication	Experimental observations for the different study parts were replicated in 6-11 mice. Each mouse was an independent observation, enabling replication of the results across mice.
Randomization	Mice were randomly allocated to the different groups, no selection was made prior to the experiments.
Blinding	Investigators were not blind to the type of the experiment and animal group. Blinding was not relevant to this study because to avoid any possible influence of the observer, scientist performing the experiment was not present in the experimental room during any behavior tests. Mice were placed in the automated maze before the start of recording (or optogenetic perturbation), and were removed only after the end of the experiment, all data was collected and analyzed automatically.

Reporting for specific materials, systems and methods

We require information from authors about some types of materials, experimental systems and methods used in many studies. Here, indicate whether each material, system or method listed is relevant to your study. If you are not sure if a list item applies to your research, read the appropriate section before selecting a response.

Materials & experimental systems

n/a	Involved in the study	
☒	☐	Antibodies
☒	☐	Eukaryotic cell lines
☒	☐	Palaeontology and archaeology
☐	☒	Animals and other organisms
☒	☐	Clinical data
☒	☐	Dual use research of concern
☒	☐	Plants

Methods

n/a	Involved in the study	
☒	☐	ChIP-seq
☒	☐	Flow cytometry
☒	☐	MRI-based neuroimaging

Animals and other research organisms

Policy information about studies involving animals; ARRIVE guidelines recommended for reporting animal research, and Sex and Gender in Research

Laboratory animals	Experiments were performed on 43 male C57BL/6 mice and 4 male transgenic mice Ai148(TIT2L-GC6f-ICL-tTA2)-D (Nr. 030328, The Jackson Laboratory) for the miniscope experiments, all aged 6–8 weeks at the first use. Animal housing was organized by the Laboratory Animal Services Center (LASC) of the University of Zurich (www.lasc.uzh.ch).<br>No field collected samples were used in this study.
Wild animals	No wild animals were used in this study.
Reporting on sex	We report on the sex of the animals used in this study in the Methods section:<br>"Experiments were performed on 34 male C57BL/6 mice and 4 male transgenic mice Ai148(TIT2L-GC6f-ICL-tTA2)-D (Nr. 030328, The Jackson Laboratory) for the miniscope experiments, all aged 6–8 weeks at the first use. "<br>"We used male mice only for practical reasons and because only one T-maze setup was available. We do not expect sex differences regarding the investigated WM mechanisms, which however will need to be tested in a separate study."
Field-collected samples	n/a
Ethics oversight	Experimental procedures were conducted in accordance with the guidelines from the Veterinary Office of Switzerland and were approved by the Zurich Cantonal Veterinary Office.

Note that full information on the approval of the study protocol must also be provided in the manuscript.

Plants

Seed stocks	n/a
Novel plant genotypes	n/a
Authentication	n/a
